# Age and co-morbidities as independent risk factors of infections leading to hospital admission in the last year of life among the elderly: A retrospective registry-based study

**DOI:** 10.48101/ujms.v129.10504

**Published:** 2024-03-13

**Authors:** Linda Björkhem-Bergman, Torbjörn Schultz, Peter Strang

**Affiliations:** a Department of Neurobiology, Care Sciences and Society (NVS), Division of Clinical Geriatrics, Karolinska Institutet, Huddinge, Sweden; b Palliative Medicine, Stockholms Sjukhem, Stockholm, Sweden; c Department of Oncology-Pathology, Karolinska Institutet, Stockholm, Sweden

**Keywords:** Elderly, age, aging, infections, co-morbidities, frailty, sex-and-gender, immunity, sociodemographic factors

## Abstract

**Background:**

The immune system declines with age, but the impact of chronological age may be affected by sex, co-morbidities, and sociodemographic factors.

**Objective:**

The article aims to study infections associated with hospital admission in the elderly in their last year of life and the impact of age, sex, co-morbidities, and sociodemographic factors.

**Method:**

A retrospective study based on registry data covering all care visits in Stockholm Region, Sweden, for 7 years was conducted. All deceased subjects with at least one hospital admission with infection as the main diagnosis in the last year of life were compared with subjects with no such admission. Subjects were categorized into three different age-groups 65–79, 80–89, and 90 years and above. Co-morbidity was measured by the Charlson Comorbidity Index (CCI) and sociodemographic factors were assessed using the ‘Mosaic-system’. Subjects living in nursing homes were analyzed separately. Uni- and multivariable logistic regressions were conducted.

**Results:**

Of the 55,238 subjects in the study population, 14,192 (26%) had at least one hospital admission due to infection in the last year of life. The risk of having a severe infection increased with age, adjusted odds ratio (OR): 1.30 (1.25–1.36), and 1.60 (1.52–1.69) for the age-groups 80–89 and ≥ 90 compared to the age-group 65–79. The most important factor for infection was a high co-morbidity score; adjusted OR: 1.75 (1.68–1.82). Male sex and living in a less affluent area were weaker risk factors for infections.

**Conclusion:**

Chronological age and co-morbidities are independent risk factors of infections associated with hospital admission in the last year in life while male sex and sociodemographic factors have less impact.

## Introduction

It is well-known that the immune system declines with age. Older individuals are more susceptible to infections and often become more ill from various infections. The reason behind this is not fully understood but many believe that this has to do with an impaired immune response over time. That elderly have a weaker immune response to new viral infections became very clear during the COVID-19 pandemic, where age was the strongest risk factor for becoming seriously ill or dying from the disease (1). New studies indicate that the elderly also respond less well to COVID-19 vaccination, which is believed to be partly related to the fact that antibodies are not produced as efficiently (2).

Even before the pandemic, we knew that an outdated immune response against, for example, influenza has important consequences as this can lead to bacterial superinfection and an increased risk of dying from, for example cardiovascular diseases (3).

However, it seems that some older individuals have a better ability than others to create an effective immune response against different forms of infections. This has raised the question of why and in what way the immune system deteriorates in the elderly – and whether it is directly linked to chronological age or whether other factors such as co-morbidities and frailty are more important.

Frailty is a state of accelerated biological aging where the body gradually loses its ability to adapt to physical, psychological, and social stressors (4, 5). Both comorbidity, as measured by Charlson Comorbidity Index (CCI) (6) and frailty, as measured by the ICD-10-based Hospital Frailty Risk Score (HFRS), were independently associated with COVID-19 deaths in cancer (7).

During the COVID-19 pandemic it was evident that male sex was a strong risk factor for severe illness and death as a result of COVID-19 compared to being female (6, 8). Interestingly, females seem to also have a better outcome for other viral and bacterial infections (9, 10). Previous studies have shown that both the innate and adaptive immune responses are generally stronger in females than males and that females also have a better response to vaccinations (11). As a consequence, females are also more susceptible to inflammatory and autoimmune diseases (11).

The aim of the present study was to map the prevalence of severe infections leading to hospital admission in the last year before death in the elderly in different age-groups from 65 years and above and evaluate possible sex-differences. More specifically, we wanted to test the hypothesis that the risk of having a severe infection in end-of-life increases with higher chronological age, independently of co-morbidity and sociodemographic factors, and that men have a higher risk of severe infections compared to women. To this end, we conducted a population-based study on all deceased individuals in the Stockholm area over 7 years with the possibility to study age- and sex-differences, adjusted for sociodemographic factors and co-morbidities.

## Method

### Study cohort

This was a retrospective study based on registry data from the administrative VAL database of the Stockholm’s region’s central data warehouse. Each clinic and care unit in Region Stockholm must report each patient visit to the VAL database and their pay from the Region (formerly: county council) is based on this data. Thus, the data are close to complete with few missing values.

In order to study infections leading to hospital admission in the elderly in the last year of life, data were collected for all deceased individuals 65 year of age or above during 2015–2021 living in the county of Stockholm, an area with approximately 2.3 million inhabitants. Subjects living in nursing homes were excluded from the main cohort since it is a population with special needs and where the treatments of infections may also be different. In the nursing homes, the residents have easier access to nurses and physicians and some infectious treatments can be imparted in the nursing home – without admission to the hospital. Thus, nursing home residents were analyzed separately.

### Main outcome

The outcome measures were infections as the main diagnosis for hospital admission at any time during the least year of life. This was defined as having at least one episode of the following International Statistical Classification of Disease version 10 (ICD-10) codes as main diagnoses for the hospital admission: A00-A99 (excluding A81.0 and A81.2), B00-B89 (excluding B18), B99, G00-G07, H00.0, H60.0, H60.3, H66, H70, I00-I01, I30.1, J00-J06, J09-J18, J20-J22, J32, J34.0, J36, J40-J42, J44, J85-J86, K57, K61, K63.0, K65.0, L00-L08, L97, M00, M46.3, M46.5, M86, N10, N13.6, N15, N30 (excluding N30.4), N34.0, N34.1, N41.0, N41.2, N41.3, N45, N70, N71.1, N76.8, N76.4, N76.0, T81.4, T82.7, T83.5, T83.6, T84.5, T84.6, and T84.7. The outcome was binary: yes or no. For explanations of the ICD-codes see Supplementary file 1.

We did not include cases with Covid-19, that is ICD-code U07.1 och U07.2 since the aim was not to study how age affected the outcome in Covid-19. However, since the pandemic probably affected the frequency of admissions to hospitals in general, we also calculated the frequency for each year and the years during the Covid-19 pandemic (2020 and 2021) was compared with the non-pandemic years (2015–2019).

A separate analysis was conducted for nursing home residents. In addition, a separate analysis of the main cohort in the last 3 months in life was conducted.

### Variables

In order to study the infection burden in the different age groups the cohort was divided into the age-groups of 65–79, 80–89, and 90 years or above. Since 65 years old is the threshold for being allowed for geriatric care in Sweden, we choose this threshold. Possible confounding factors were collected and adjusted for. This included sex, sociodemographic index measured by mosaic described further, and comorbidities measured by CCI. Both uni- and multivariable analyses were performed.

In order to study sex differences, the same analyses were performed but divided into men and women separately.

### Measure of co-morbidity

Charlson Comorbidity Index is a ICD-10 based construct and a measure of comorbidity. CCI is based on 19 ICD-10 diagnostic codes, where different codes add between 1 and 6 points to the index (12). A score of 0–2 is assessed as a low degree of co-morbidity and above 2 as a high burden of co-morbidities. Infections are not part of the score and thus CCI was assessed as suitable for this study, as it was strongly associated with COVID-19-related cancer deaths in a recent study (6).

In register data covering large populations, data on frailty measured with the widely used clinical frailty score (CFS) is not accessible (13). Instead, other tools for assessment of frailty, based on ICD-10 codes, such as HFRS have been developed (14). Since HFRS also includes infections as part of its score, that is infections add scores to the severity of frailty, we assessed that this tool, after pilot testing, was less suitable to use in the present study. Thus, we only used the CCI index in this study, as a proxy for frailty, although frailty and comorbidity are different constructs (12).

### Measure of sociodemographic factors

Mosaic is a system that divides a county or city into different groups of socioeconomic areas and can be used for studies where sociodemographic factors may have an impact (15, 16). It showed to be predictive during the first wave of the COVID-19 pandemic (16). Mosaic is based on information of median income, education, lifestyle, and living arrangements in a specific living area. The Stockholm Region is divided into 1,300 small areas (containing 1,500–1,800 inhabitants), and each area is classified as Mosaic 1, 2, or 3, where Group 1 corresponds to the most affluent areas. In the current study, we merged the group Mosaic 1 and 2 (affluent and middle-class areas) and compared them with Mosaic 3, that is less affluent areas.

### Statistical analysis

Descriptive statistics are presented as means and standard deviations (SD). Differences between groups were assessed using Chi^2^-test for categorical variables and t-test for continues variables. Univariable logistic regression analysis were followed by multivariable logistic regressions models. For the comparison between different age-groups, adjustments were made for sex, CCI, and Mosaic. For the comparison between men and women, adjustments were made for age-groups, CCI, and Mosaic. Odds ratios (OR) were calculated, with 95% Confidence Intervals (CI) for each comparison.

In the logistic regression models, the youngest age-group, being women, and belonging to the highest sociodemographic group, that is Mosaic group 1–2 and having the ‘healthiest’ CCI-index, that is CCI 0-2, were chosen as reference groups.

As a measure of goodness of fit for binary outcomes in our multiple logistic regression models, we calculated C-statistic (equivalent to the area under the receiver operating characteristic curve). A C-statistic value of 0.5 indicates that the model is no better than chance at making a prediction of membership in a group and a value of 1.0 indicates that the model perfectly identifies those within a group and those not.

### Ethics

This study was approved by a decision of the Regional Ethical Review Board in Stockholm 2017 approving that all data from the VAL database on deceased patients were approved to be used for research studies (Dnr 2017/1141-31). All data were pseudonymized before analysis.

No written informed consent could be obtained since only deceased patients were included.

## Results

In the VAL-database, 97,708 deceased patients 65 years or older were identified in the Stockholm Region between 2015 and 2021 of which 55,238 had been living at home and 42,425 had been living in nursing homes at some time-point during the last year of life.

### Infections leading to hospital admission in the last year of life

The study population for the main analysis was thus 55,283, 25,810 women, and 29,473 men. The median age was 82 years (range: 65–107). The demographic data of the study population are presented in Table 1. Women were generally older in the last year of life; they had fewer co-morbidities and were living in less-affluent areas compared to men (Table 1). In the study population, 14,192 (26%) persons had at least one hospital admission during their last year of life. The most common infections leading to hospital admission were pneumonia (ICD-codes: J189 and J159) which constituted 30% of all infections. Exacerbation of Chronic Obstructive Pulmonary Disorder (COPD, ICD-codes: J44.1 and J44.9) amounted for 16%, whereas sepsis (ICD-code A419) and tubulointerstitial nephritis (ICD-code N10.9) constituted 5% each.

**Table 1:** Demographic data of deceased subjects 65 years or older.

Variables	Total *n* = 55,283	Women *n* = 25,810	Men *n* = 29,473	*P*
**Age-groups**				
65–79 years (*n*)	26,672 (48%)	11,188 (43%)	15,484 (53%)	< 0.001
80–89 years (*n*)	19,100 (35%)	9,002 (35%)	10,098 (34%)	< 0.001
90 years and above (*n*)	9,511 (17%)	5,620 (22%)	3,891 (13%)	< 0.001
**Co-morbidity**				
Low CCI: 0–2, %	22,332 (40%)	10,966 (42%)	11,366 (39%)	< 0.001
High CCI: > 2, %	32,951 (60%)	14,844 (58%)	18,107 (61%)	< 0.001
**Sociodemographic area**				
Mosaic 1–2	35,155 (64%)	16,044 (62%)	19,111 (65%)	< 0.001
Mosaic 3	20,128 (36%)	9,766 (38%)	10,362 (35%)	< 0.001
**Infections leading to hospitalization,** *n* (%)	14,192 (26%)	6,562 (25%)	7,630 (26%)	0.21

All deceased subjects 65 years and older in the Stockholm Region 2015–2021. Nursing homes residents were not included in the cohort. Values show amount (*n*) and % within parenthesis. Differences between men and women were assessed using Chi^2^-test. Mosaic 3 = less affluent area.

The risk of having an infection leading to hospital admission increased with age and chronological age was also a significant risk factor after adjustments were made for co-morbidity, sex, and sociodemographic factors (Table 2). However, the strongest risk-factor for infection was co-morbidity as measured by CCI, both in the uni- and multivariable analyses. Living in a less affluent area was also a risk factor for infections both in the uni- and multivariable analyses, although the impact of this variable was less pronounced. Male sex showed a weak, but statistically significant, association with risk of infections in the adjusted model. When women and men were analyzed separately the same pattern appeared, that is chronological age and co-morbidity were independent risk factors in both men and women (data not shown).

**Table 2:** Logistic regression of hospital admission during the last year of life, with infection as the main diagnosis of deceased elderly, 65 years and older in the Stockholm Region living at home (i.e. not nursing homes).

Variable	*n*	Univariable analysis		Multivariable analysis*	
		OR (95% CI)	*P*	OR (95% CI)	*P*
**Sex**					
Women	25,810	Ref.		Ref.	
Men	29,473	1.03 (0.99–1.07)	0.21	1.05 (1.01–1.09)	0.03
**Age groups** 65–79 years 80–89 years 90 years or more	26,672 19,100 9,511	Ref. 1.29 (1.24–1.35) 1.49 (1.41–1.57)	< 0.001 < 0.001	Ref. 1.30 (1.25–1.36) 1.60 (1.52–1.69)	< 0.001 < 0.001
**Co-morbidity** CCI 0–2 CCI > 2	22,332 32,951	Ref. 1.70 (1.63–1.77)	< 0.001	Ref. 1.75 (1.68–1.82)	< 0.001
**Sociodemographic area** Mosaic 1–2 Mosaic 3	35,155 20,128	Ref. 1.05 (1.01–1.09)	0.03	Ref. 1.07 (1.02–1.11)	0.03

Mosaic 3 = less affluent area,

* c statistic was 0.59.

### Infections during the last 3 months

Next, hospital admissions due to infections during the last 3 months in life were analyzed separately. A similar pattern appeared, that is older age and a high co-morbidity index were significantly associated with higher risk of hospitalization due to infections (data not shown). Being male and living in a less affluent area was also associated with a slightly increased risk of hospitalizations due to infections.

### Infections in the last year of life in nursing homes residents

A separate analysis was also conducted in nursing homes residents since this group of elderly differs from those living at home. In line with the main analysis, a high co-morbidity index was an independent risk factor for infections that required hospitalization (Table 3). However, increasing age was a significant factor for *not* being admitted to hospital for infections. In addition, male sex was significantly associated with hospital admission due to infections, while sociodemographic factors were not.

**Table 3:** Logistic regression of hospital admission the last year of life with infection as the main diagnosis in nursing home residents 65 years and older in the Stockholm Region.

Variable	*n*	Univariable analysis		Multivariable analysis*	
		OR (95% CI)	*P*	OR (95% CI)	*P*
**Sex**					
Women Men	26,055 16,370	Ref. 1.86 (1.78–1.95)	< 0.001	Ref. 1.53 (1.45–1.60)	< 0.001
**Age groups** 65–79 years 80–89 years 90 years or more	7,101 16,963 18,361	Ref. 0.81 (0.77–0.87) 0.55 (0.52–0.59)	< 0.001 < 0.001	Ref. 0.88 (0.82–0.94) 0.70 (0.66–0.75)	< 0.001 < 0.001
**Co-morbidity** CCI 0–2 CCI > 2	26,675 15,750	Ref. 3.01 (2.95–3.23)	< 0.001	Ref. 2.82 (2.69–2.95)	< 0.001
**Sociodemograpic area** Mosaic 1–2 Mosaic 3	27,349 15,076	Ref. 1.04 (0.99–1.09)	0.15	Ref. 1.01 (0.96–1.06)	0.66

Mosaic 3 = less affluent area

* c statistic was 0.58.

### Hospital admission due to infections – differences during the years

Since the COVID-19 pandemic might influence the hospital admissions during 2020 and 2021 we conducted a separate analysis to study differences between the years. There was a significant decrease in number of hospital admissions due to infections in 2020 (25%) and in 2021 (23%) compared to 2015–2019 (27%) (*P* < 0.01). For nursing homes residents, the decrease was even more pronounced, in 2020 (18%) and 2021 (22%) compared to 2015–2019 (25%) (*P* < 0.001).

## Discussion

In this study, we could confirm our hypothesis that high chronological age is an independent risk factor for having an infection leading to hospital admission in the last year in life. However, co-morbidity was the strongest independent risk factor for having infections that required hospitalization. Adjustment for sociodemographic factors and sex did not affect the risk. Still, the increased risk for infections in relation to age and co-morbidities can be considered as only moderate as judged by the OR values and the goodness of fit statistics, that is the c-statistic values.

Previous studies have shown that elderly persons are generally more susceptible to infections and often have poorer outcomes of infections compared to younger individuals (1, 2, 17). Interestingly, our results also show that chronological age is an independent risk factor, also when adjustment was made for co-morbidities. This strengthens the hypothesis that the immune function deteriorates with age – regardless of other factors. Our results further strengthen the fact that there is also a gradient of impairment with chronological age from 65 years and above.

We could also confirm previous results showing that a high CCI index, score 3 and above, is an independent risk factor for infections and poorer outcome (6, 18).

Studies during the COVID-pandemic showed that male sex was an independent risk factor for having a more severe infection or to die from the disease (6, 8). In addition, other studies have shown that men have a higher risk of infections (9–11). Our results show that being male was a weak risk factor for hospital admission due to infections during the last year in life for people living in their own homes. However, for people living in nursing homes, male sex was an independent and important risk factor for being admitted to acute hospitals due to infections, regardless of age, co-morbidity, and sociodemographic factors. However, this is not necessarily a measure of a higher susceptibility to infections but might reflect a general tendency to admit male nursing home residents to acute hospitals, despite similar conditions. This was also recently shown in a cohort of more than 30,000 nursing home residents (19).

In this study, sociodemographic factors measured with Mosaic had some impact on the risk of having an infection – but other factors were more important. This is in contrast to previous studies on COVID-infections in the same population (Region Stockholm) where living in less-affluent areas was an important risk factor for severe infections and death as a result of COVID-19 (16). This might be explained by the fact that people living in these areas were the most unprotected from COVID-19 when vaccines and protective equipment were missing during the first year of the pandemic. They were taxi drivers, bus drivers, workers in restaurants and in places where they were generally more exposed to the COVID-19 virus than people living in affluent and middle-class areas.

The number of hospital admissions due to infections (except for COVID-19) decreased during the pandemic, 2020 and 2021. During these years most people, especially elderly, avoided going to the hospital and the emergency departments due to restrictions. The findings with a pronounced reduced number of admissions due to infections among nursing home residents during the pandemic years are in line with clarified national recommendations regarding prioritizing intensive care under extraordinary conditions (20). In summary, the importance of considering biological age and its impact on patient benefit to ensure that the strained resources were used for the patients who were expected to benefit the most.

We also studied data for the last 3 months in life separately in order to evaluate if there were factors that were especially important during the last months in life. In the 3-month data, co-morbidity and high chronological age were also the most important risk factors for hospital admission, as in the main analysis.

Notably, the data from nursing homes showed a completely opposite pattern regarding age where younger age was associated with hospital admission due to infection. We cannot explain these findings, but it indicates that there is an inequality between younger and older residents at nursing homes, where nursing home care is supplemented with hospital care to a greater degree for younger residents. However, further investigations are needed before firm conclusions can be drawn.

The data in this study cover the whole population of deceased subjects 65 years and older in the Stockholm region. Although there might be regional differences, we think that the results might be generalizable for elderly in Sweden and in other countries as well with similar socioeconomics.

### Strengths and limitations

The major strength of this study is that we have a large cohort with consecutive data, with almost no missing data.

A limitation is that we only know that the subjects were admitted to hospital due to infections, but not the outcome of the infections. For example, we cannot know whether men had longer hospital stays or died more frequently due to infections – only that they were treated more often. Another limitation is that we could not study how frailty affected the risk of infections since all frailty tools developed for registry data comprise infections as a variable. Notably, we attempted to use the HFRS-score (14) but after pilot-testing we assessed this as a less suitable tool for this study, although HFRS-score was a highly significant variable in univariable comparisons (data not shown). Although co-morbidity co-variates with frailty to some extent – prognostic tools developed specifically for frailty seem to be more sensitive than CCI (21, 22).

To conclude, this study shows that chronological age and having a high co-morbidity index were independent risk factors for having an infection leading to hospital admission during the last year of life.

## Supplementary Material


