# Supplementary material for: Age and co-morbidities as independent risk factors of infections leading to hospital admission in the last year of life among the elderly: A retrospective registry-based study
Source: Ups J Med Sci. 2024 Mar 13;129:10.48101/ujms.v129.10504. doi: 10.48101/ujms.v129.10504 (PMC10989213; doi:10.48101/ujms.v129.10504)
Supplement: Supplementary file 1 [file UJMS-129-10504-s1.docx]

**Supplementary file 1**

Explanations of the ICD-Codes:

- A00-A99 (excluding A81.0 and A81.2), B00-B89 (excluding B18) - Certain infectious and parasitic diseases
- G00-G07 – Infectious diseases of the central nervous system
- H00.0, H60.0, H60.3, H66, H70 – Infectious disease in the eye and ear
- I00-I01, I30.1 – infectious disease in the circulation
- J00-J06, J09-J18, J20-J22, J32, J34.0, J36, J40-J42, J44, J85-J86, - Infectious disease in the respiratory system
- K57, K61, K63.0, K65.0 – Infectious disease in the digestive system
- L00-L08, L97 - Infectious disease in the skin and subcutaneous tissue
- M00, M46.3, M46.5, M86 - Infectious disease in the musculoskeletal system and connective tissue
- N10, N13.6, N15, N30 (excluding N30.4), N34.0, N34.1, N41.0, N41.2, N41.3, N45, N70, N71.1, N76.8, N76.4, N76.0 - Infectious disease in the genitourinary system
- T81.4, T82.7 , T83.5, T83.6, T84.5, T84.6, T84.7 Infectious disease due to external causes
